# Immature Platelet Fraction as a Potential Biomarker of Dysregulated Thrombopoiesis in Philadelphia-Negative Myeloproliferative Neoplasms

**DOI:** 10.3390/jcm15031140

**Published:** 2026-02-02

**Authors:** Ivan Zekanovic, Tina Marketin, Martina Moric Peric, Drazen Zekanovic, Ante Vulic, Marija Milos, Anamarija Bogic, Marta Marcinkovic, Petra Grbic Pavlovic, Margareta Radic Antolic, Josip Knezevic, Ljiljana Jurlina, Ana Boban

**Affiliations:** 1 Department of Internal Medicine, General Hospital Zadar, 23000 Zadar, Croatia; 2 Department of Hematology, University Hospital Centre Zagreb, 10000 Zagreb, Croatia; 3 Department of Laboratory Diagnostics, University Hospital Centre Zagreb, 10000 Zagreb, Croatia; 4 Faculty of Pharmacy, University of Mostar, 88000 Mostar, Bosnia and Herzegovina; 5 Department of Laboratory Diagnostics, General Hospital Zadar, 23000 Zadar, Croatia; 6 School of Medicine, University of Zagreb, 10000 Zagreb, Croatia

**Keywords:** Ph^−^ MPN, IPF, JAK2, platelets

## Abstract

**Background/Objectives**: Thrombotic events represent the leading cause of morbidity and mortality in patients with Philadelphia chromosome-negative myeloproliferative neoplasms (Ph^−^ MPNs), particularly in those aged > 60 years. The immature platelet fraction (IPF) reflects the proportion of newly released, reticulated, highly reactive platelets and has emerged as a marker of thrombopoietic activity in various prothrombotic conditions. **Methods**: We prospectively measured IPF in 45 patients with newly diagnosed Ph^−^ MPNs (24 with essential thrombocythemia, 13 with polycythemia vera, 5 with MPN-unclassified, and 3 with primary myelofibrosis) and 27 controls without MPN. **Results**: IPF was significantly higher in patients with Ph^−^ MPN than in controls (median 27 vs. 10.9, *p* < 0.0001). Within the MPN cohort, IPF values differed significantly across subtypes (*p* = 0.027), being highest in essential thrombocythemia and primary myelofibrosis, intermediate in unclassified MPN, and lowest in polycythemia vera. Patients older than 60 years exhibited higher IPF independently of platelet count (*p* = 0.021). No significant difference was observed between JAK2V617F-positive and -negative cases. **Conclusions**: These results indicate that IPF captures accelerated and dysregulated thrombopoiesis characteristics of Ph^−^ MPNs and may provide additional insight into subtype-specific biology and age-related prothrombotic risk beyond conventional complete blood count parameters.

## 1. Introduction

The Philadelphia chromosome-negative myeloproliferative neoplasms (Ph^−^ MPNs) are clonal neoplastic disorders of the myeloid hematopoietic stem cells (HSCs). These disorders are classified into polycythemia vera (PV), essential thrombocythemia (ET), primary myelofibrosis (PMF), and rarer entities such as chronic neutrophilic leukemia, chronic eosinophilic leukemia, and unclassified MPN(MPN-U) [1]. The main mutations in the gene for Janus kinase 2 (JAK2V617F), the thrombopoietin receptor (MPL), and calreticulin (CALR) are present in over 90% of patients with MPN and promote the activation of the JAK-STAT signaling pathway, resulting in cytokine-independent proliferation of one or more myeloid lineages [2].

Thrombotic events can occur at the time of diagnosis in up to 20% of patients with Ph^−^ MPN and are the most common cause of morbidity and mortality in patients with MPN. Both arterial (myocardial infarction and ischemic stroke) and venous events (deep-vein thrombosis, pulmonary embolism, and splanchnic vein thrombosis) are markedly increased in patients with MPN compared with age-matched populations [3]. A retrospective analysis of patients with MPNs from the Swedish Cancer Registry reported that at 3 months after diagnosis, patients with PV had an approximately 3- and 13-fold higher risk of arterial thrombosis and venous thrombosis, respectively, compared with controls matched for age and sex [3]. In a prospective study from the German SAL-MPN registry that included 455 patients with Ph-negative MPN, 33.6% of patients experienced a vascular event. The most frequent events were deep vein thrombosis (31.5%), acute coronary syndrome (27.7%), stroke (19.3%), and splanchnic vein thrombosis (15.2%) [4]. Ph^−^ MPNs are the most frequent underlying cause of non-cirrhotic Budd Chiari syndrome (~40% of cases) and are identified in approximately one-third of patients with portal vein thrombosis; the JAK2 V617F mutation is present in 80–87% of these patients [5]. Moreover, cardiovascular risk factors and predisposition to thrombosis have been shown to have a positive correlation in Ph^−^ MPN [6].

Despite extensive research, the precise mechanisms driving the prothrombotic state in MPN remain incompletely understood. Thrombosis arises from complex interactions among quantitative and qualitative abnormalities of blood cells, endothelial activation, inflammation, and alterations in the coagulation cascade [7]. The release of neutrophil extracellular traps (NETs), a meshwork of DNA fibers comprising histones and antimicrobial proteins which are extracellular net-like structures composed of modified DNA and associated enzymes such as myeloperoxidase (MPO) and neutrophil elastase among other proteases and lysozymes, has been described as a possible scaffold for thrombus formation [8], with studies suggesting that NET components are present and may contribute to the procoagulant state in these disorders [9] and that platelets and JAK2-V617F neutrophils interact to enhance NET formation [10]. In addition to elevated platelet counts, abnormal platelet function significantly contributes to the prothrombotic state in myeloproliferative neoplasms (MPNs) [11]. Patients with ET and PV exhibit enhanced ADP-induced platelet aggregation and increased thrombin generation, with the most pronounced alterations observed in JAK2 V617F-positive individuals and, paradoxically, in some patients receiving aspirin therapy [12]. An increase in platelet count can be seen in all subtypes of MPN but is particularly present in ET. The level of thrombocytosis has not been proven to significantly correlate with thrombosis risk in essential thrombocythemia [13].

Although the risk of thrombotic complications is evaluated using the current scoring systems, such as in polycythemia vera, which includes age and history of thrombosis, or the IPSET score in essential thrombocythemia, which additionally includes the presence of the JAK2 mutation [14], there remains the question of whether there is room for other markers of hypercoagulability that are easily accessible in everyday clinical practice.

Immature (reticulated) platelets are newly released from megakaryocytes, are larger, contain more dense granules and residual mRNA, and exhibit greater thrombotic potential than mature platelets [15,16].

Elevated immature platelet fraction (IPF) predicts worse outcomes in acute coronary syndromes, ischemic stroke, and after coronary stenting, and correlates with aspirin and clopidogrel resistance [17,18]. Small studies on MPNs have reported increased IPF values, but systematic comparison across subtypes and age groups, and driver mutation status is lacking [19]. The measurement of IPF can help us differentiate platelet destruction conditions, such as ITP or TTP, in comparison to hypoproliferative states [20]. Immature platelets can be measured using supravital dye staining (e.g., new methylene blue) on blood films, or with fluorescent dyes (e.g., thiazole orange) and flow cytometry [21]. Immature platelet fraction measurements obtained using Sysmex automated hematology analyzers show correlation with reticulated platelet counts determined by standard flow cytometry [22].

We therefore conducted a prospective study to characterize IPF in patients newly diagnosed with Ph^−^ MPN, compare it with non-MPN controls, and explore its variation according to subtype, age, and JAK2V617F status in order to evaluate its potential relevance as a marker of hypercoagulability.

## 2. Materials and Methods

### 2.1. Study Design

This prospective observational study was performed at General Hospital Zadar and University Hospital Centre Zagreb, Croatia, between March 2024 and October 2025. All adult patients undergoing diagnostic bone marrow examination for suspected Ph^−^ MPN were eligible. The exclusion criteria comprised previously known MPN, active malignancy, recent surgery/trauma (<4 weeks), acute infection, pregnancy, or any condition known to secondarily elevate IPF (e.g., recent major bleeding).

Diagnoses of PV, ET, PMF, and MPN-unclassified were established in accordance with the International Consensus Classification of Myeloid Neoplasms and Acute Leukemias [1]. Patients in whom MPN was excluded after a complete work-up constituted the control group. Peripheral blood samples were collected at the time of diagnostic bone marrow biopsy. IPF was measured on Sysmex XN-1000 analyzers (Kobe, Japan) using fluorescent flow cytometry and proprietary RNA-binding polymethine dye. Excellent reproducibility for IPF measurement on the Sysmex XN-1000 was achieved at multiple IPF levels, with coefficients of variation of 3.6% for IPF = 5.3%, 2.0% for IPF = 21.7%, and 1.1% for IPF = 66.2%. Additional laboratory parameters included complete blood count, LDH, urea, creatinine, and D-dimer.

This study was approved by the Ethics Committees of both institutions and conducted in accordance with the Declaration of Helsinki.

### 2.2. Statistics

Descriptive statistics were used for the summary presentation of patient characteristics. According to Shapiro–Wilk’s test, the data were not symmetrically distributed; thus, nonparametric statistical tests were used. Continuous variables were compared with the Mann–Whitney U test. Continuous variables were compared across groups using the Kruskal–Wallis test. All analyses were performed using the MedCalc Statistical Software version 23.4.8. (MedCalc Software Ltd., Ostend, Belgium). Significant *p*-values were set at <0.050 for all presented analyses.

## 3. Results

### 3.1. Patients Characteristics

Forty-five patients with newly diagnosed Ph^−^ MPN and 27 controls were enrolled.

Among patients with MPN, 24 (53%) patients were diagnosed as having essential thrombocythemia, 13 (28%) patients with polycythemia vera, 5 (11%) with unclassified MPN, and 3 (6%) with primary myelofibrosis. The control group comprised 27 individuals in whom Ph^−^ MPN was excluded. There were 19 (42%) females in the Ph^−^ MPN group and 8 (29%) in the control group (Table 1).

### 3.2. Results

Patients with Ph^−^ MPN were significantly older than controls (median 65 vs. 50 years) and had higher platelet counts (median 572 × 10^9^/L vs. 238 × 10^9^/L, *p* < 0.0001) and LDH (median 226 vs. 178 U/L, *p* < 0.001). Leukocyte, neutrophil, erythrocyte counts, hemoglobin, and hematocrit did not differ significantly between the groups.

**Figure 1:**
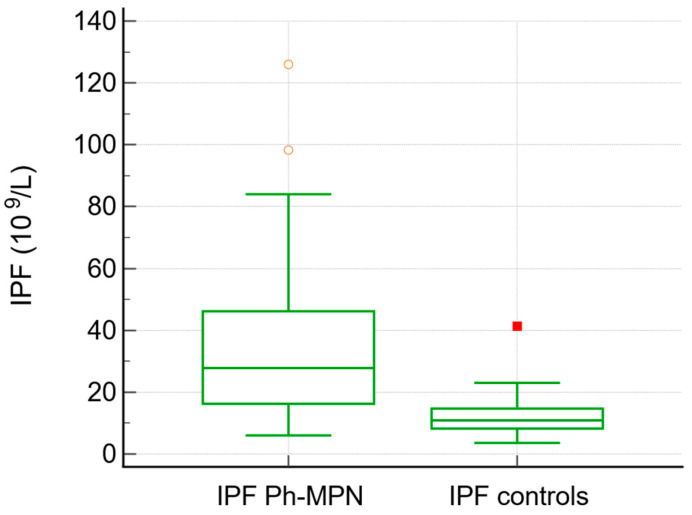
Box-and-whisker plot. IPF values were higher in patients with Ph^−^ MPN compared to the control group.

JAK2V617F was positive in 75% of patients with MPN overall (92% of PV, 70% of ET, 66% of PMF, and 80% of unclassified MPN). The IPF values were higher in patients with Ph^−^ MPN (median 27, range 6–126) in comparison to the control group (median 10.9, range 3.6–41, *p*< 0.0001) (Table 2, Figure 1). Neutrophil-to-lymphocyte ratio (NLR) was significantly higher in patients with MPN compared with controls (median 3.68 vs. 2.68, *p* = 0.013). Similarly, D-dimer levels were elevated in the MPN group (median 0.50 vs. 0.33, *p* = 0.048).

No differences were observed in IPF values between JAK2-positive and JAK2-negative patients with MPN (median 26 vs. 40.7, *p* = 0.25). However, higher values of hematocrit were found among JAK2-positive patients (median 0.47 vs. 0.41, *p* = 0.032). (Table 3).

When comparing the patients with Ph^−^ MPN in the age group older than 60 years, we found values of IPF (median 38.8, range 10.2–126) which were higher compared to those in patients with Ph^−^ MPN who were younger than 60 years (median 23.5, range 6–50, *p* = 0.021), but we did not find any difference in the platelet count between these two age groups (median 572 vs. 604, *p* = 0.84) (Table 4).

To compare the differences between the subgroups of MPN, a Kruskal–Wallis test was performed. It showed significant differences in IPF between the MPN subgroups (*p* = 0.027). The median IPF values were highest in essential thrombocythemia and primary myelofibrosis, intermediate in unclassified MPN, and lowest in polycythemia vera. The median IPF values were as follows: ET 41.6, PMF 46.2, MPN-unclassified 25, and PV 17.2 × 10^9^/L. Platelet counts followed the expected pattern (highest in ET and lowest in PV), whereas granulocyte counts were highest in PMF and unclassified MPN.

**Figure 2:**
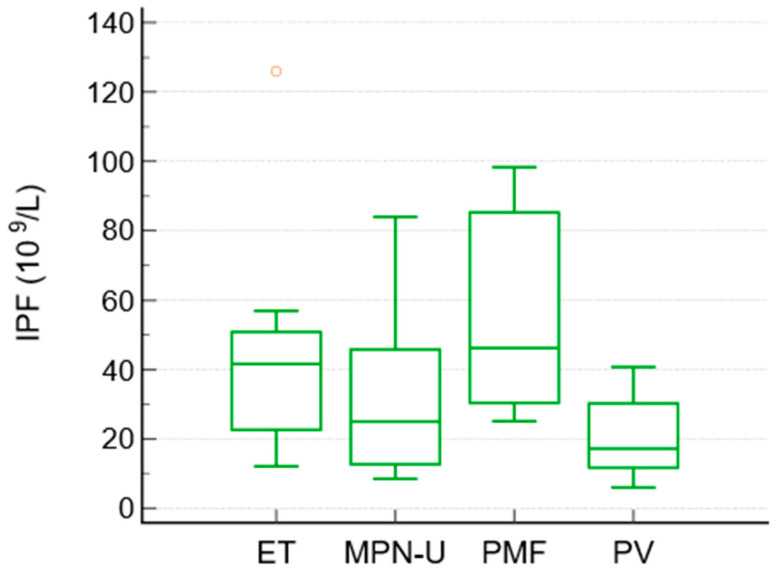
Box-and-whisker plot. Distribution of IPF among subtypes of Ph^−^ MPN.

No other pairwise differences between the subtypes reached statistical significance (Table 5, Figure 2).

## 4. Discussion

Our study provides a comprehensive analysis of immature platelet fraction in Ph^−^ MPNs. The nearly threefold elevation in IPF in patients with Ph^−^ MPN compared with carefully selected non-MPN controls confirms markedly accelerated thrombopoiesis, even at disease presentation and before cytoreductive therapy.

IPF has shown prognostic relevance in cardiovascular and cerebrovascular disorders [17,18], but its role in myeloproliferative neoplasms as a marker of hypercoagulability is not completely understood.

The pathophysiological basis is likely multifactorial. Clonal megakaryocytes in Ph^−^ MPNs exhibit hypersensitivity to thrombopoietin and other cytokines, increased ploidy, and proplatelet formation independent of physiological feedback [23]. The resulting release of young, RNA-rich platelets with heightened metabolic and prothrombotic activities (increased expression of P-selectin, GPIIb/IIIa, and tissue factor, and enhanced response to ADP, thrombin, and shear stress) contributes directly to the hypercoagulable states [24].

Interestingly, the analysis stratified by age revealed that patients with Ph^−^ MPN older than 60 years had significantly higher IPF values than younger patients (median 41.6 vs. 23.6, *p* = 0.0202). This could suggest that age-related factors, including marrow remodeling and comorbidities, may further amplify dysregulated platelet production in MPN. Importantly, this increase in IPF occurred independently of platelet count, highlighting that conventional platelet numbers may underestimate underlying thrombopoietic activity. Our data could support the concept that IPF reflects qualitative platelet dysfunction better than absolute count alone.

We also identified differences in IPF among MPN subtypes. The Kruskal–Wallis test showed significant heterogeneity, with post hoc analyses demonstrating higher IPF levels in ET and MF compared to PV. In PMF, ineffective hematopoiesis, inflammatory cytokine signaling, and early extramedullary hematopoiesis may further promote the release of immature platelets, while in ET, clonal megakaryocytic proliferation drives increased platelet production [25,26]. The highest platelet count was observed in ET, followed by PMF, and the highest granulocytes values were observed in PMF and unclassified MPN. Among JAK2-positive patients, IPF values differed significantly across the MPN subtypes, with PV showing the lowest values compared with ET and MF.

These findings may reflect the distinct pathophysiology of each MPN subtype. Our findings suggest that IPF could provide additional insight into the biology of platelet production across MPN subtypes and patient subgroups. Elevated IPF in Ph^−^ MPN could support its role as a marker of increased thrombopoietic activity, while the influence of age emphasizes the importance of integrating IPF into broader clinical and biological assessments. Importantly, the lack of correlation with platelet counts could indicate that IPF may capture aspects of platelet dynamics that routine hematology parameters fail to reflect. Previous studies have demonstrated that both JAK2V617F mutational status and hydroxyurea therapy independently influence immature platelet parameters in essential thrombocythemia and polycythemia vera, which could explain the favorable effect of hydroxyurea therapy on MPN outcome as well as the thrombotic risk [27]. Increased neutrophil-to-lymphocyte ratio (NLR) was also observed in patients with MPN and could represent either myeloproliferation itself [28] or the degree of chronic inflammation [29], while higher D-dimer levels, particularly in patients older than 60 years, may indicate persistent coagulation activation and endothelial dysfunction characteristic of the prothrombotic MPN phenotype [30].

From a clinical perspective, IPF is measured automatically on widely available hematology platforms without additional cost or phlebotomy. If future longitudinal studies confirm that high IPF identifies patients at increased thrombotic risk, especially within the same conventional risk category, it could refine stratification and guide intensity of cytoreduction or antithrombotic prophylaxis.

Several limitations must be acknowledged. The sample size was relatively small, particularly for the PMF subgroup, which included only three patients, and for the unclassified MPN group, which included only five patients; this limitation restricts the generalizability of the findings within these categories.

The cross-sectional design also precludes conclusions about the prognostic role of IPF. Furthermore, we did not assess correlations between IPF and thrombotic outcomes, which represent a clinically relevant endpoint in MPN. Nevertheless, this study provides novel comparative data on IPF across MPN subtypes and highlights important biological patterns that merit further exploration.

## 5. Conclusions

IPF is significantly elevated in Philadelphia chromosome-negative myeloproliferative neoplasms and shows subtype- and age-specific patterns. These findings could highlight IPF as a readily available marker of dysregulated thrombopoiesis that may complement conventional hematological parameters. Further studies are warranted to determine whether IPF can improve thrombotic risk stratification in Ph^−^ MPNs.

## Figures and Tables

**Table 1:** Demographic data and patient characteristics.

Variable	Ph^−^ MPN (*n* = 45)	Control Group (*n* = 27)
ET	24 (53%)	
PV	13 (28%)	
MPN unclassified	5 (11%)	
PMF	3 (6%)	
Age	65 (24–87)	50 (21–76)
Sex, female	19 (42%)	8 (29%)
JAK2	33 (73%)	

ET = essential thrombocythemia, PV = polycythemia vera, PMF = primary myelofibrosis, JAK2 = Janus Kinase 2.

**Table 2:** Patients with Ph^−^ MPN compared with the control group.

Variable	Ph^−^ MPN (*n* = 45)	Control Group (*n* = 27)	*p* Value *
IPF × 10^9^/L (median, range)	27 (6–126)	10.9 (3.6–41)	**<0.0001**
Platelets × 10^9^/L (median, range)	572 (141–1292)	238 (133–690)	**<0.0001**
Erythrocytes × 10^12^/L (median, range)	5.23 (2.8–10.1)	5.64 (3.57–6.98)	0.913
Leukocytes × 10^9^/L (median, range)	9.4 (4.93–24.9)	9.35 (5.70–21.50)	0.521
Hematocrit % (median, range)	0.46 (0.26–0.73)	0.49 (0.32–0.57)	0.332
Granulocytes × 10^9^/L (median, range)	6.20 (2.32–38.59)	5.64 (2.70–18.04)	0.220
Lymphocytes × 10^9^/L (median, range)	2.02 (0.79–5.49)	2.28 (1.35–3.75)	**0.037**
Neutrophil to lymphocyte ratio (NLR)	3.68 (0.85–16.28)	2. 68 (1.18–5.18)	**0.013**
Creatinine μmol/L (median, range)	78 (44–125)	80 (50–206)	0.443
LDH U/L (median, range)	226 (86–509)	178 (81–592)	<0.0001
D-dimer mg/mL (median, range)	0.5 (0.19–6.21)	0.33 (0.18–2.46)	**0.048**

* Statistically significant *p* values are bolded and set at <0.050. The Mann–Whitney U test was used. IPF = immature platelet fraction, LDH = lactate dehydrogenase.

**Table 3:** JAK2-positive Ph^−^ MPN compared with the JAK2-negative Ph^−^ MPN.

Variable	Ph^−^ MPN JAK2 Positive (*n* = 33)	Ph^−^ MPN JAK2 Negative (*n* = 12)	*p* Value *
IPF × 10^9^/L (median, range)	26 (6–126)	40.7 (8.5–98.3)	0.254
Platelets × 10^9^/L (median, range)	557 (141–1134)	652 (296–1292)	0.501
Erythrocytes × 10^12^/L (median, range)	5.4 (3.7–8.6)	4.6 (2.88–10.15)	0.056
Leukocytes × 10^9^/L (median, range)	9.45 (4.93–24.90)	10.20 (5.7–17.7)	0.877
Hematocrit % (median, range)	0.47 (0.34–0.63)	0.41 (0.26–0.73)	**0.032**
Granulocytes × 10^9^/L (median, range)	6.38 (3.75–38.5)	5.78 (2.32–12.29)	0.449
Neutrophil-to-lymphocyte ratio (NLR)	3.68 (1.58–16.28)	2.48 (0.85–7.68)	0.161
D-dimer mg/mL (median, range)	0.58 (0.10–6.21)	0.47 (0.28–0.8)	0.694
Creatinine μmol/L (median, range)	79 (54–125)	69 (44–101)	0.369
LDH U/L (median, range)	225 (86–509)	240 (122–439)	0.635

* Statistically significant *p* values are bolded and set at <0.050. The Mann–Whitney U test was used. IPF = immature platelet fraction, LDH = lactate dehydrogenase.

**Table 4:** Results across age groups of patients with Ph^−^ MPN.

Variable	Ph^−^ MPN > 60 Years (*n* = 29)	Ph^−^ MPN < 60 Years (*n* = 16)	*p* Value *
IPF × 10^9^/L (median, range)	38.8 (10.2–126)	23.5 (6–50)	**0.0218**
Platelets × 10^9^/L (median, range)	572 (141–1264)	604 (296–1292)	0.849
Erythrocytes × 10^12^/L (median, range)	5.23 (2.88–10.15)	5.21 (4.43–8.81)	0.766
Leukocytes, × 10^9^/L (median, range)	10 (4.93–23)	8.6 (6.2–24.9)	0.399
Hematocrit % (median, range)	0.46 (0.26–0.73)	0.46 (0.36–0.66)	0.803
Granulocytes × 10^9^/L (median, range)	6.5 (2.32–38.59)	5.34 (3.96–20.94)	0.367
Neutrophil-to-lymphocyte ratio (NLR)	3.68 (0.85–16.2)	2.92 (1.67–8.1)	0.420
D-dimer mg/mL (median, range)	0.68 (0.2–6.21)	0.31 (0.19–0.7)	**0.004**
Creatinine μmol/L (median, range)	85 (44–125)	72.5 (54–118)	0.114
LDH U/L (median, range)	225 (86–509)	233 (122–455)	0.872

* Statistically significant *p* values are bolded and set at <0.050. The Mann–Whitney U test was used. IPF = immature platelet fraction, LDH = lactate dehydrogenase.

**Table 5:** IPF values in subtypes of Ph^−^ MPN.

Variable	ET (*n* = 24)	PV (*n* = 13)	PMF (*n* = 3)	MPN-U (*n* = 5)	*p* Value *
IPF × 10^9^/L (median, range)	41.6 (12–126)	17.2 (6–40.7)	46.2 (25–98)	25 (8.5–84)	**0.027**
Platelets × 10^9^/L (median, range)	675 (320–1292)	422 (277–848)	496 (301–638)	436 (141–544)	**0.0009**

* Statistically significant *p* values are bolded and set at <0.050. The Kruskal–Wallis test was used. IPF = immature platelet fraction, ET = essential thrombocythemia, PV = polycythemia vera, PMF = primary myelofibrosis, MPN-U = unclassified MPN.

## Data Availability

The data presented in this study are available on request from the corresponding author (Ivan Zekanovic) due to privacy reasons.
